# Interaction of Glide Dislocations with Extended Precipitates in Mg-Nd alloys

**DOI:** 10.1038/s41598-018-20629-1

**Published:** 2018-02-23

**Authors:** Zhihua Huang, John E. Allison, Amit Misra

**Affiliations:** Department of Materials Science and Engineering, University of Michigan, Ann Arbor, United States

## Abstract

The unit processes of precipitate-dislocation interaction in dilute Mg-Nd alloys are elucidated through *in situ* indentation experiments in TEM. Results suggest that pinned dislocations can glide along the broad facets of extended β_1_ precipitates, a common strengthening phase in Mg- rare earth (RE) alloys. A dislocation-theory based analysis suggests that the shape, spacing and orientation (with respect to the glide plane) of β_1_ precipitates may favor glide of pinned dislocations along interfaces as opposed to the classical mechanism of bowing and looping around the precipitate.

## Introduction

As the lightest structural metal, magnesium is becoming increasingly important for light weighting strategies as fuel efficiency is of great concern to industries such as automotive^1,2^ and aerospace^3^. For many magnesium alloys, precipitation hardening is an important mechanism for impeding dislocations on the basal glide planes. In particular, precipitates that are aligned parallel to the prism planes, so-called prismatic precipitates, have been shown to be effective in pinning basal dislocations^4,5^. Commercial alloys such as WE54, WE43 and Mg-RE (RE represents rare earth element) utilize the prismatic β_1_ precipitate as a strengthening phase^6–8^. The β_1_ phase has an ordered structure D0_3_ (space group $Fm\bar{3}m$, ${a}_{{\beta }_{1}}$ = 0.74 nm) with a composition of Mg_3_RE^9^, and is generally regarded as non-shearable by basal slip in Mg matrix^10^.

For non-shearable precipitates, the increase in critical resolved shear stress (CRSS), τ_Orowan_, may be estimated by the Orowan mechanism of dislocation bowing and looping around an array of point obstacles^11–14^. The equation is given as

1 $${\tau }_{Orowan}=\frac{Gb}{2\pi \lambda \sqrt{1-v}}ln\frac{{d}_{p}}{{r}_{0}}$$

where λ is the inter-particle spacing in the slip plane, d_p_ and r_0_ are outer and inner cut-off radii for calculation of dislocation energy, G is shear modulus of the matrix phase, b is the magnitude of the Burgers vector, and υ is Poisson’s ratio. However, in practical alloys such as Mg-RE alloys, precipitates may have finite sizes and a non-spherical or non-equiaxed shape. Certain corrections have been made to include precipitate shape and orientation effects in equation (1). Stark *et al*.^15^ worked on the self-stress effect of dislocation caused by different precipitate-dislocation configurations. Nie *et al*.^5^ on the other hand, studied the effect of precipitate distribution and morphology on inter-particle spacing. Several attempts to simulate dislocation-precipitate interaction have also been made by dislocation dynamics (DD) and phase-field^16,17^ in which the essence of interaction is still based on Orowan strengthening. Although there are many attempts to correct or simulate the Orowan mechanism, the dynamic processes for dislocations overcoming the precipitates remain unclear. Direct visualization of dislocation-precipitate interactions will be critical in developing physics-based models of unit mechanisms that can also be inputs for dislocation dynamics simulation^18^ with predictive capability.

In the present work, *in situ* indentation in the TEM has been used to study the unit processes of precipitate-dislocation interaction in a dilute Mg-Nd alloy. The observed interactions are rationalized using a dislocation-theory based analysis.

## Results

**Figure 1:**
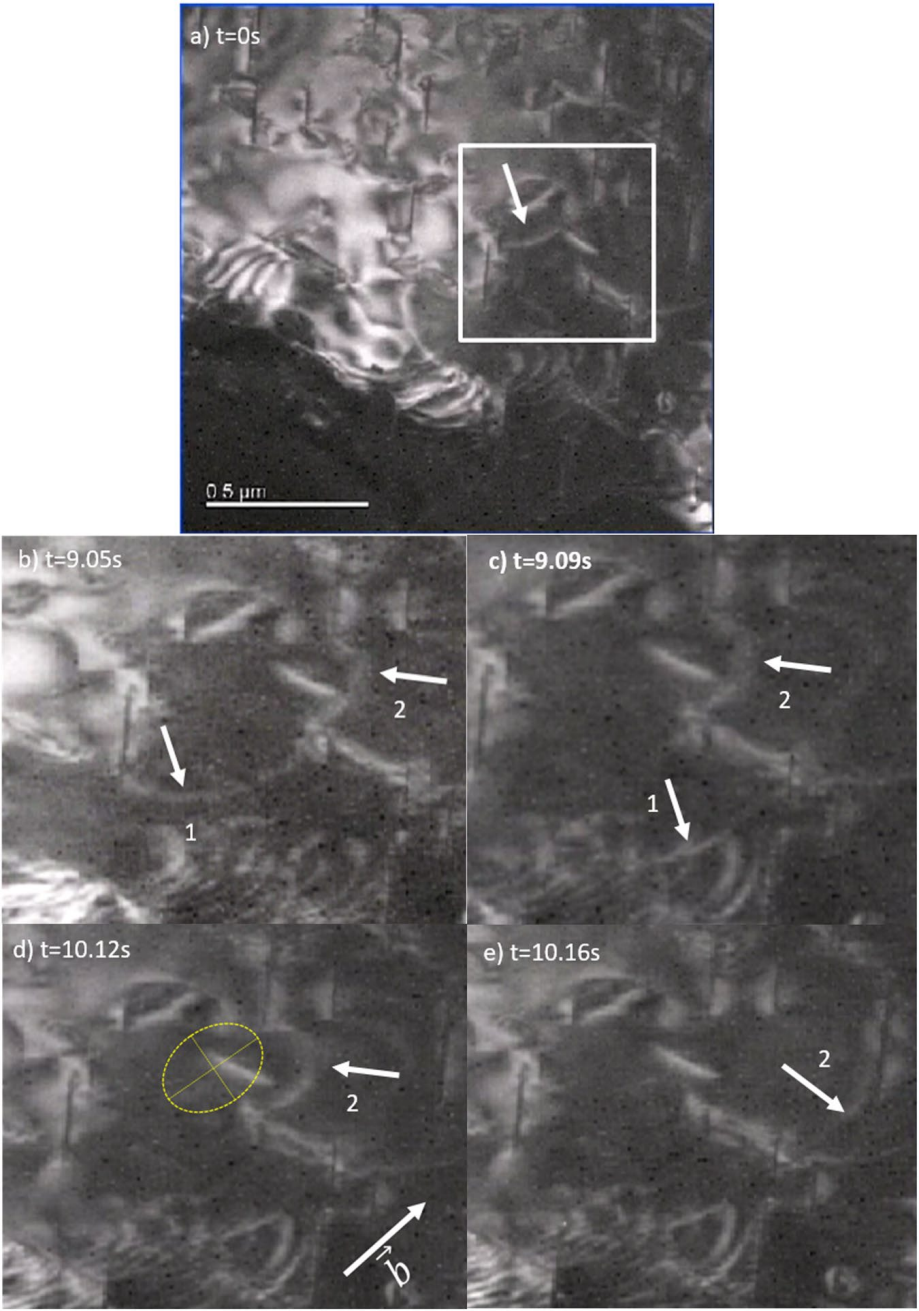
TEM images of dislocation glide on the basal plane. The region in Fig. 1b,c,d,e are from the marked region in Fig. 1a. The time interval between (**b**) and (**c**), (**d**) and (**e**) is very short (one frame 0.04 s) recording the dislocation propagation for both segments. Frame (**d**) shows an elliptical shape of the bowed dislocation segment 2.

Two scenarios are presented in this section viewing the precipitate-dislocation interactions from either <0001> or $< \bar{1}2\bar{1}3 >$ orientations. The β_1_ precipitates have a lath morphology and are parallel to the $(10\bar{1}0)$ habit plane. The orientation relationship between β_1_ precipitates and matrix is $\,{[10\bar{1}0]}_{m}\parallel {[\bar{1}12]}_{p}\,$and ${(0001)}_{m}\parallel {(110)}_{p}$. The precipitates have a high aspect ratio and are approximately 200 nm along both length $[\bar{1}2\bar{1}0]$ and height [0001] dimensions but only 10 nm in thickness. In the over-aged aging condition studied (9 hours at 250 °C), the β_1_ precipitates are prone to be semi-coherent. In order to have the best contrast, both bright field and dark field diffraction conditions were used. For the (0001) view plane, dark field imaging was used and dislocations appear as bright lines in section 2*.a* (Supplementary video 2.a) whereas for the $\,(\bar{1}2\bar{1}3)$ view plane, bright field imaging was used and dislocations appear as dark lines in section 2*.b* (Supplementary video 2.b). The *in situ* experiments were supplemented with post mortem analysis that indicated that the predominant slip systems were <a> Burgers vectors on basal planes, that is also the slip system reported to have the lowest CRSS in magnesium^19^. Bowed dislocations also tend to elongate along the Burgers vector to increase the screw component to minimize its energy, resulting in an elliptical shape such as the bowed segment (marked as 2) in Fig. 1d.

### Dislocation Glide along Interfaces

Figure 1 shows the behavior of a single dislocation interacting with an extended β_1_ precipitate. The glide dislocation is observed to be pinned and then eventually bypass the precipitates. The selected area is tilted to be near to the basal plane. The studied dislocation segment is marked with an arrow in Fig. 1(a). With increasing stress during the indentation process, the initial dislocation starts to glide and reaches a new pinned configuration in Fig. 1b.

**Figure 2:**
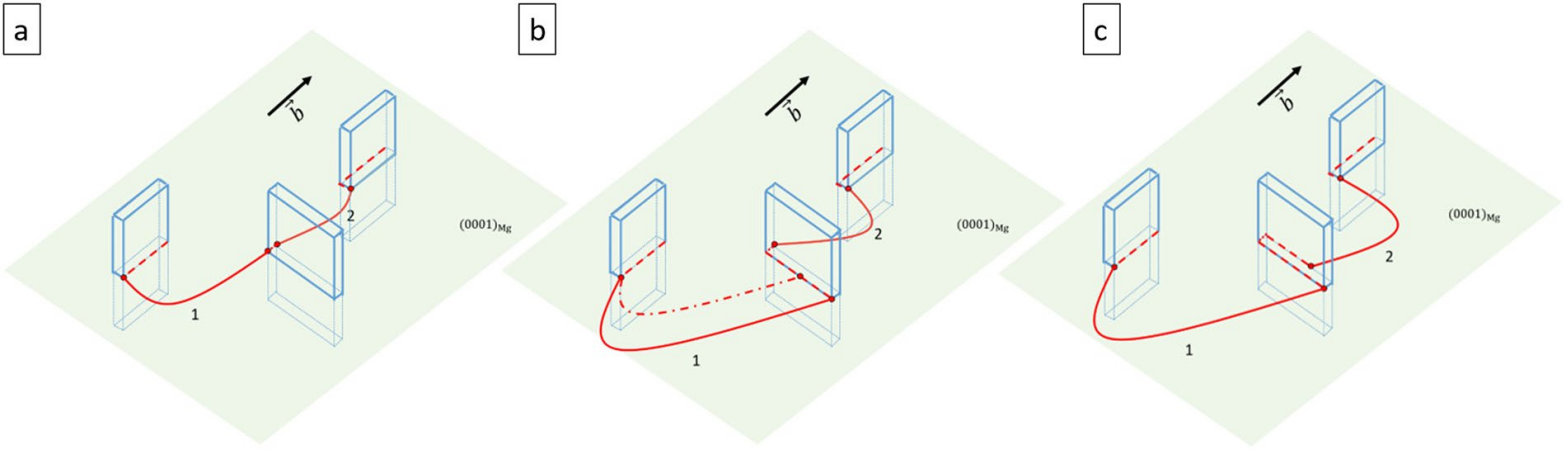
Schematics of precipitate-dislocation interactions depicting progression of the experimental images shown in Fig. 1. β_1_ precipitates are oriented perpendicular to the basal plane depicted as plates with broad facets. Dislocations are constrained in the basal plane and the interfacial dislocations are represented by red dashed lines.

The pinned dislocation segment in Fig. 1(b) has been divided into part 1 and 2. It is noticeable that part 1 starts to glide (from Fig. 1b to c) by moving the right end on the precipitate facet. Figure 2 shows the corresponding schematic view of the observed dislocation-precipitate interaction. In Fig. 2a to b, the pinned segments 1 and 2 continue bowing as stress increases to a point when the right end of segment 1 moves along the precipitate interface. The dashed dotted line in Fig. 2(b) portrays an intermediate position of segment 1 between 9.05 s to 9.09 s in the *in situ* experiment. In this study, we define the dislocation configuration at the onset of interfacial glide as the critical condition. In order to maintain the continuity of the dislocation, the glide along the interface is expected to create additional dislocation line lengths at the interface, which are represented as red dash lines in Fig. 2. From Fig. 1b to c, once the interface glide initiates, the right end of segment 1 transitions to the departure side of precipitate in one frame (0.04 s). The mobility of the free end to glide along the precipitate interface is significantly higher than the bowing process leading to a fast transition from one pinned configuration to another one. In considering Figs 1 and 2, it should be noted that interfacial glide does not occur simultaneously for segments 1 and 2. Although the pinned segments 1 and 2 are formed from one single original dislocation, the bowing and interfacial glide behavior are independent from each other. This indicates that the pinned dislocation behavior depends on the segment between two pinned points rather than the whole dislocation, which is one of the basic assumptions in the following model.

### Orientation-dependent effect

**Figure 3:**
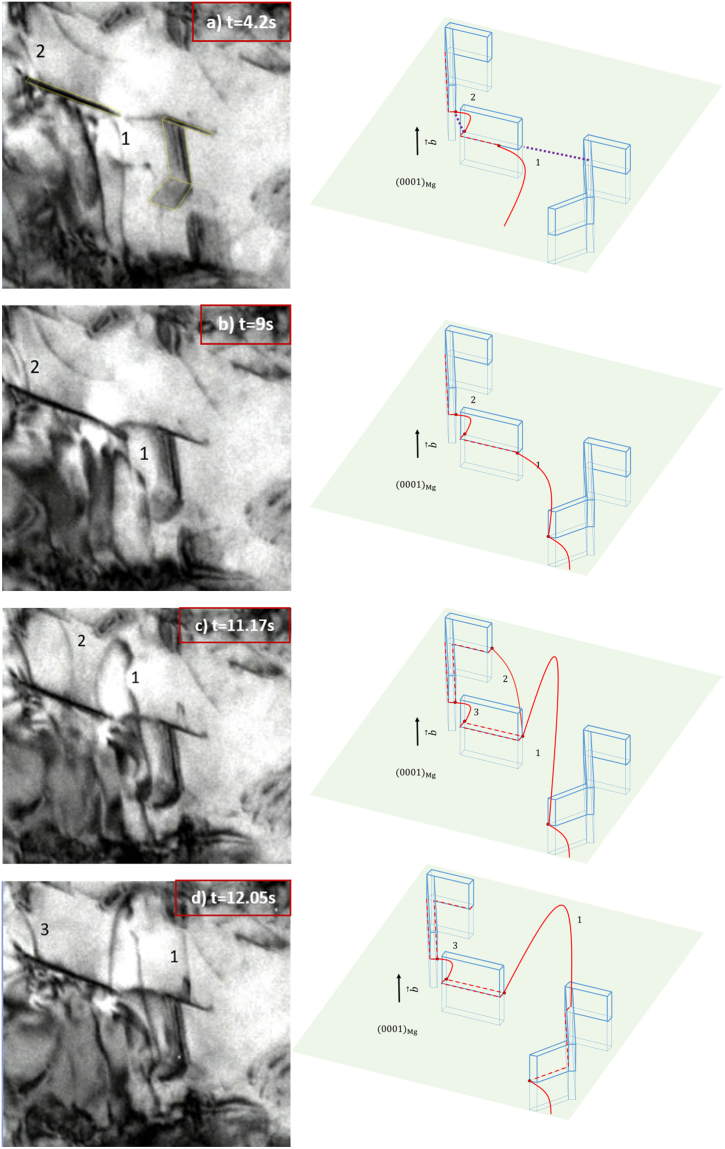
TEM images of motion of dislocations on basal (0001) plane thread through two parallel precipitates. Schematics in the right column correspond to the recorded frame at left. The viewing direction $[\bar{1}2\bar{1}3]$ of images relative to schematics is shown in schematic (**a**). The precipitates of interest are also outlined in (**a**). Initial dislocation line direction is represented as purple dotted line in (**a**). After segment 2 glides out of view, a following dislocation segment 3 is pinned in (**c**) and (**d**).

In Fig. 3, a foil region is examined that is oriented near to $(\bar{1}2\bar{1}3)$. Thus the foil plane lies 30° from the basal axis so that the precipitates are observed in a slant view. In section 2*.a*, dislocations initially interact with the narrow edge of the β_1_ precipitate, followed by the formation of interfacial dislocations on the broad facets as interfacial glide occurs. In this *in situ* video, these interactions happen with the broad facets and display the different dislocation behaviors. Figure 3 shows two different precipitate-dislocation configurations at the same time. Two pinned dislocations are studied in this case: 1) A dislocation (marked as 1) is observed to glide in from the left bottom of the image and makes contact with the broad facet of the precipitates. 2) A dislocation (marked as 2) is observed to be pinned at the narrow edge of the precipitate near the left top of the image. In general, the two segments do not have to be in the same basal slip plane but in the schematic, are shown in the same plane for convenience. In Fig. 3(a), a purple dotted line is used as a reference to connect the two pinned nodes for Segment 2 and mark the orientation of the initial dislocation line. The main difference between the two pinned segments is the angle between the initial dislocation line direction and the broad precipitate facet. We define this angle as orientation angle. For segment 2, its initial dislocation line is about 30° away to the precipitate long axis, whereas the initial dislocation line of segment 1 is nearly parallel to it.

Segment 2 in Fig. 3 behaves similar to the dislocation in Fig. 1 in that it bows against the narrow edge and initiates interfacial glide when it reaches the critical condition. As in Fig. 3(c), after interfacial glide occurs at precipitates interfaces, segment 2 is pinned at the departure side. Without strong bowing, segment 2 is able to glide out of view at Fig. 3(d), which indicates a weak detachment force. In contrast, the behavior of segment 1 is completely different. As shown in Fig. 3(a), the segment 1 first approaches the broad interface of the precipitate and creates an interfacial dislocation marked as red dashed line in the schematics. Then, it gets pinned as depicted in Fig. 3(b). When it continues to glide, it bows around two pinned nodes in Fig. 3(c) and creates interfacial dislocations at precipitate interfaces as the dotted lines represent in Fig. 3(d). Comparing these two segments, from Fig. 3(a) to (c), both segments are bowed from the pinned nodes but segment 2 is able to glide at precipitate interface, whereas no interfacial glide occurs on segment 1 after it is pinned at the position depicted in Fig. 3(d).

The *in situ* experiment shows two different bowing configurations for segment 1 and 2. Segment 1 bows much more than segment 2. Because the back stress exerted from dislocations are related to the bowing shape, the difference in bowing shape is deemed to indicate these segments have different pinning stresses. The interfacial glide of segment 2 facilitates its propagation without the need to further increase bowing under the applied stress. There is no fundamental difference between segment 1 and 2 in terms of dislocation characteristics. As mentioned above, it is the precipitate-dislocation configurations that are different and the occurrence of the proposed interfacial glide mechanism reduces the pinning effect for specific dislocation-precipitate geometries. In this video, precipitates with lower orientation angles appear to exhibit a stronger pinning effect than those with larger angles.

In summary, the different bowing configurations between these two segments gives an indication that the strengthening effect for single precipitate-dislocation interaction depends on the relative precipitate-dislocation orientation.

## Discussion

**Figure 4:**
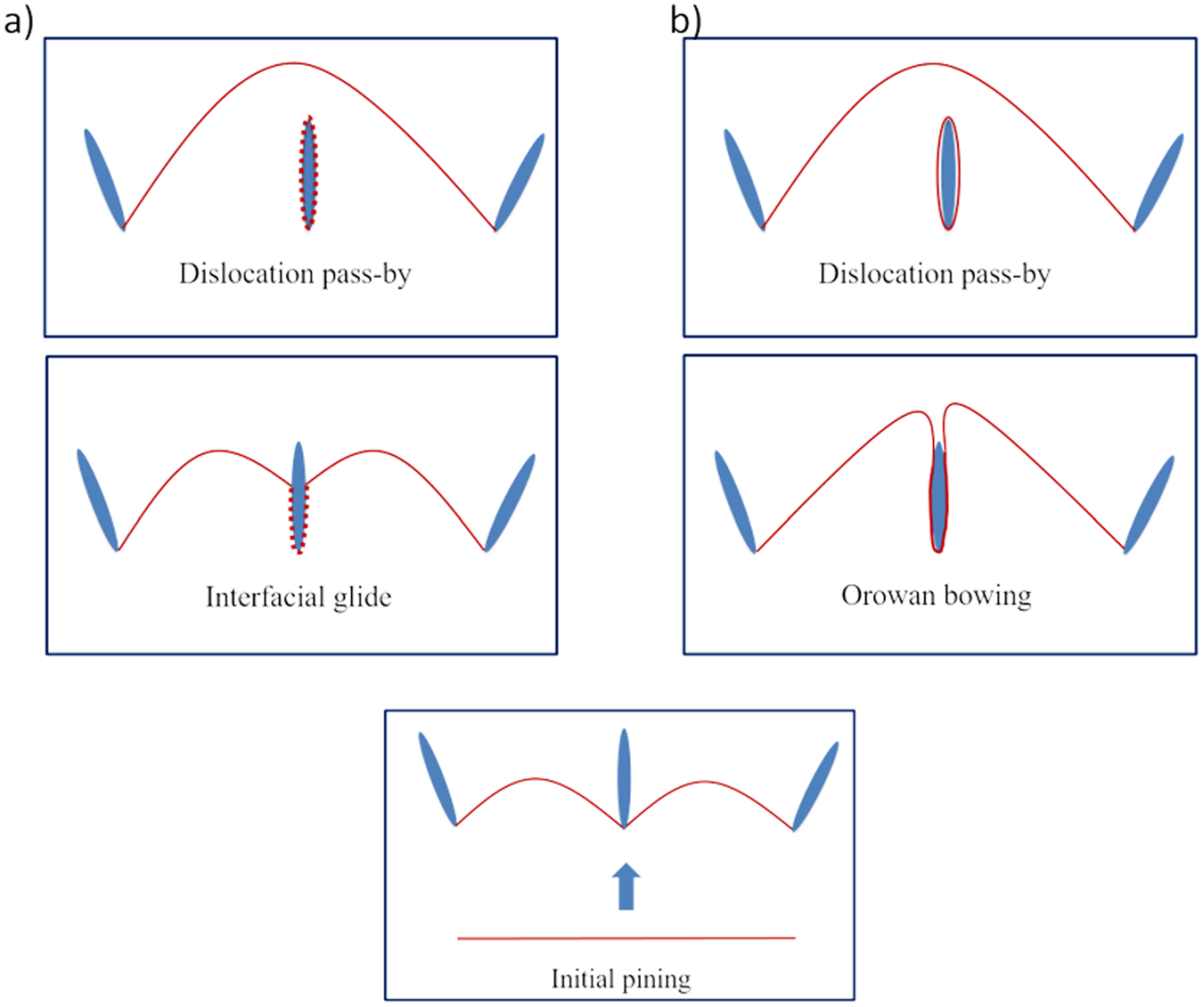
Schematic diagram of (**a**) proposed interfacial glide mechanism and (**b**) classical bowing mechanism for overcoming the given precipitate configuration. The dotted line around the center precipitate is the interfacial dislocation created by forward dislocations. Solid lines represent the initial position, critical condition, intermediate position and final position from bottom to top.

In the *in situ* straining experiment, we have observed interfacial glide at broad facet of linear extended β_1_ precipitates. Unlike the classical bowing mechanism in which the dislocation encircles the precipitate completely to form a loop, interfacial glide can create dislocations at interfaces without extensive bowing. For a given precipitate-dislocation configuration, Fig. 4 shows the difference between the two mechanisms. In both situations, dislocations are pinned at the leading edge of precipitates. However, after reaching the critical condition, interfacial glide is initiated in Fig. 4(a) showing a different sequence that occurs creating interfacial dislocations compared with the classical bowing shown in Fig. 4(b). The conditions that favor the unit process shown in 4 a) over 4 b) are analyzed below based on classical dislocation theory.

### Dislocation Theory analysis

**Figure 5:**
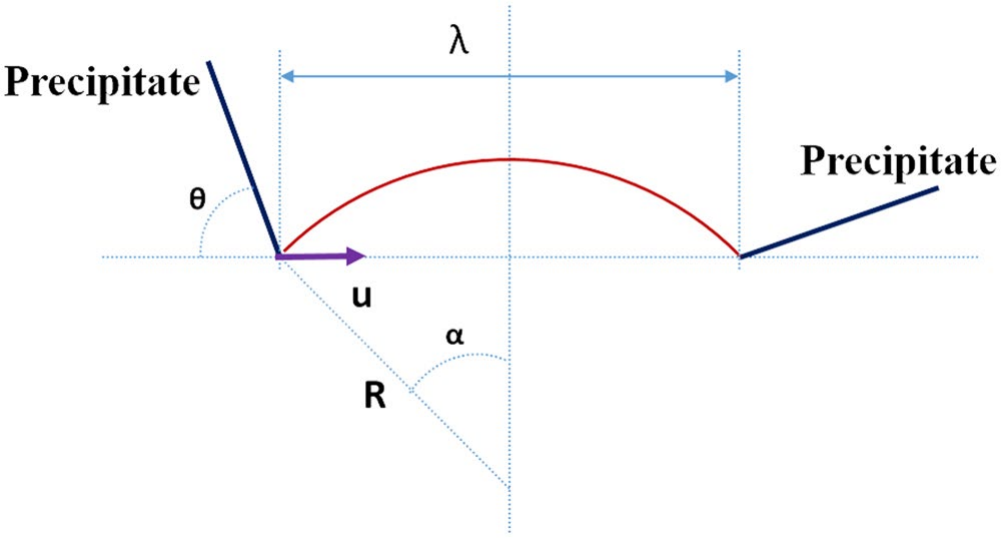
Dislocation-precipitate geometry for a pinned dislocation segment. The arrow indicates, as a reference, the initial unbowed dislocation line direction (u). For the precipitate on the left, the orientation angle θ is defined as measured clockwise from u to the trace of the broad facet of the precipitates. For the precipitate on the right, it is defined as measured counter clockwise.

In order to comprehend this phenomenon, a model based on dislocation line tension is developed, as shown schematically as in Fig. 5.

In this model, we assume that given the different θ values for the two pinning points, the initiation of glide along the interface may be favored for one point over the other. It has to be mentioned that this is a generic scenario where dislocation get pinned by two extended precipitates. We do not presume any particular orientation between precipitates in the model although a 60° or 120° angle will occur between different β_1_ precipitate variants. The conclusions are applicable for any general configuration of precipitates.

Once the dislocation begins to glide along the interface, it will lose the force equilibrium until it reaches another pinned position, normally the departure side of the precipitate. The detachment process is complicated and is not within the scope of this paper.

For the sake of mathematical simplicity, the bowed segment is treated as an arc with a radius of R. Angle α is the half of central angle for the bowed segment. For a given inter-particle spacing λ, a larger value of α shows a stronger bowing segment. We define ${\rm{x}}=\,\sin ({\alpha }^{\ast })=\frac{\lambda }{2R}$ as the bowing magnitude, ranging from 0 to 1, which is related to the strength of the pinning point, where α^*^ represents a critical value of α.

**Figure 6:**
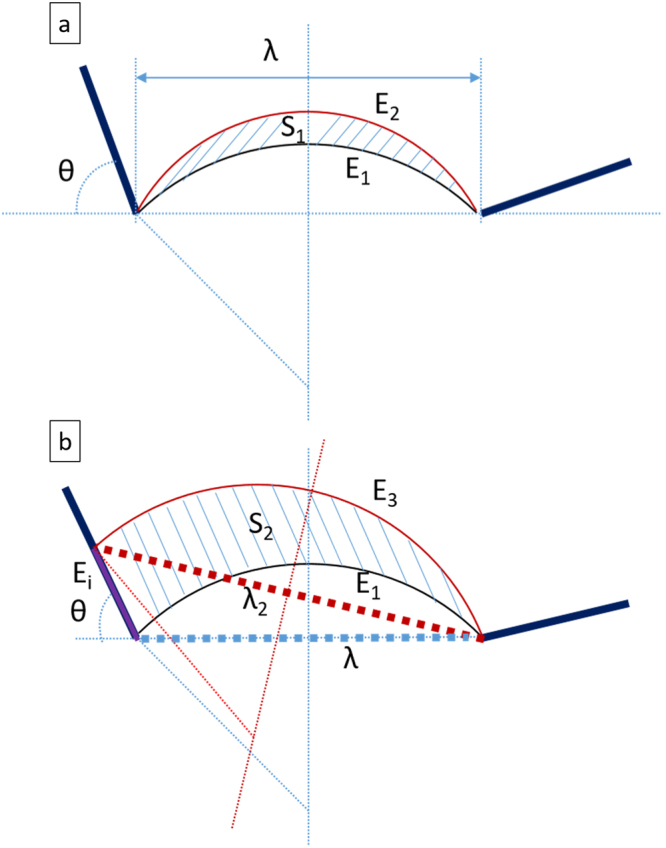
Schematic of two dislocation bowed shapes due to different energy configurations: (**a**) bowing without interfacial glide and (**b**) interfacial glide from the critical condition. The interfacial dislocation in (**b**) is represented by the purple line. This infinite small interfacial dislocation segment is exaggerated in the schematic for viewing purpose. The heavy dotted lines highlight the distance between pinned points.

Figure 6 shows two possible configurations of the pinned dislocation, Fig. 6(a) for the case of no glide along interface and Fig. 6(b) for the case of dislocation glide along an interface.

E_1_ is the dislocation energy at the critical condition. If there is no interfacial glide, Fig. 6(a), the pinned dislocation will continue bowing to a new position with an energy of E_2_. The area swept by dislocation bowing is S_1_ and is related to the plastic work done. On the other hand, if interfacial glide occurs, Fig. 6(b), after infinite short amount of time, an infinitely small interfacial dislocation segment is created with an energy of E_i_ and the new curved dislocation has an energy of E_3_. The area swept by dislocation movement is S_2_. From an energy point of view, interfacial glide can initiate only if the energy of the second configuration, Fig. 6(b), is lower than the first one, Fig. 6(a). Therefore, the energetic criterion to initiate interfacial glide is as follows:

2 $${E}_{3}+{E}_{i}-\tau b{S}_{2} < {E}_{2}-\tau b{S}_{1}$$

where τ is the Peach-Koehler stress. If the bowed dislocation is in force equilibrium at the critical condition, the external stress is equal to the back stress calculated from the dislocation.

3 $$\tau =\frac{T}{bR}=\frac{Gb}{2R}$$

where T is the line tension which is approximately $\frac{G{b}^{2}}{2}$ using a constant line tension model.

Since the bowed shape is assumed to be an arc, i.e., it is insensitive to the direction of the Burgers vector, a constant line tension model is applied so that the dislocation line energy is only dependent on dislocation length. Then the energy of E_1_ can be expressed as:

4 $${E}_{1}=\frac{G{b}^{2}}{2}(2Rarcsin\frac{\lambda \,}{2R})$$

After an incremental interfacial glide, as in Fig. 6(b), it is assumed that the new curved dislocation glides along the precipitate broad facet with no change in curvature, which gives its energy E_3_:

5 $${E}_{3}=\frac{G{b}^{2}}{2}(2Rarcsin\frac{{\lambda }_{2}}{2R})$$

It should be noticed that λ_2_ is larger than λ so that the length of the new curved dislocation is longer than the dislocation when it is in the critical condition. This leads to an increase in the energy *δE*. The energy criteria can be revised as:

6 $$\delta E+{E}_{i}+\tau b({S}_{1}-{S}_{2}) < {E}_{2}-{E}_{1}$$

The evolution from E_1_ to E_2_ is because of a change in the radius of curvature. Since the change is infinitesimal, the criterion becomes:

7 $$\delta E+{E}_{i}+\tau b({S}_{1}-{S}_{2}) < dE$$

The right side of equation (7) is a derivative of equation (4) with regard to radius R. Its expression is given as:

8 $$dE=\frac{G{b}^{2}}{2}(\frac{2\frac{\lambda \,}{2R}}{\sqrt{1-{(\frac{\lambda }{2R})}^{2}}}-2arcsin\frac{\lambda \,}{2R})dR$$

To solve *δE*,

9 $$\begin{array}{rcl}\delta E & = & {E}_{3}-{E}_{1}\\ \delta E & = & \frac{G{b}^{2}}{2}\,\ast \,2R(arcsin\frac{{\lambda }_{2}\,}{2R}-arcsin\frac{\lambda \,}{2R})\end{array}$$

Because the difference between λ_2_ and λ is infinitesimally small, the value of the expression in parentheses in equation (9) can be solved by Taylor expansion. Expanding arcsinx on x = $\frac{\lambda \,}{2R}$ yields

10 $$arcsinx=\arcsin (\frac{\lambda \,}{2R})+\frac{1}{\sqrt{1-{(\frac{\lambda }{2R})}^{2}}}(x-\frac{\lambda \,}{2R})+o{(x-\frac{\lambda }{2R})}^{2}$$

The last term of this polynomial is a higher order term that can be ignored in the following derivation. Utilizing the approximation in equation (10) and setting x = $\,arcsin\frac{{\lambda }_{2}\,}{2R}$, the equation (9) can be approximately written as

11 $$\delta E={E}_{3}-{E}_{1}=\frac{G{b}^{2}}{2}\frac{1}{\sqrt{1-{(\frac{\lambda }{2R})}^{2}}}({\lambda }_{2}-\lambda )$$

λ_2_ can be expressed as

12 $$\begin{array}{c}{\lambda }_{2}=\sqrt{{(\lambda +dlcos\theta )}^{2}+{(dlsin\theta )}^{2}}\\ {\lambda }_{2}=\sqrt{{\lambda }^{2}+2\lambda dlcos\theta +d{l}^{2}}\end{array}$$

where *dl* is the length of the interfacial dislocation, which is also an infinitesimally small value. Hence, *λ*_2_ − *λ* is infinitesimally small. The approximation of that difference is in the same manner as the previous Taylor expansion. The result can be expressed as

13 $${\lambda }_{2}-\lambda =\frac{1}{2\lambda }(2\lambda dlcos\theta +d{l}^{2})$$

Ignoring the higher order term *dl*^2^, the final expression for *δE* is

14 $$\delta E={E}_{3}-{E}_{1}=\frac{G{b}^{2}}{2}\frac{1}{\sqrt{1-{(\frac{\lambda }{2R})}^{2}}}dlcos\theta $$

**Figure 7:**
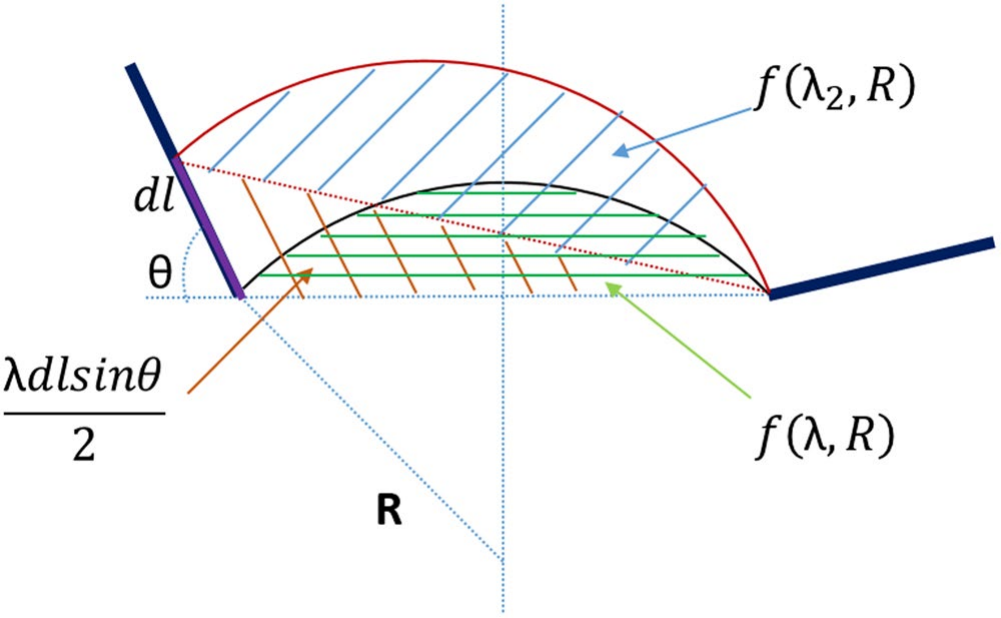
Schematic shows area of different regions. The green shaded area is the dislocation swept area before interfacial glide is initiated. The sum of orange and blue shaded area is the swept area caused by bowing and interfacial glide.

To solve S_1_ and S_2_, we come up with a function defining the green shaded area in Fig. 7.

The function can be expressed as

15 $$S=f(\lambda ,R)={R}^{2}\arcsin (\frac{\lambda \,}{2R})-\frac{\lambda \,}{2}\sqrt{{R}^{2}-{(\frac{\lambda }{2})}^{2}}$$

The expression for S_1_ is basically the derivative of *f*(*λ*, *R*) with respect to radius R.

16 $${S}_{1}=\frac{\partial f}{\partial R}(-dR)=(-\frac{2\lambda R}{\sqrt{4{R}^{2}-{\lambda }^{2}}}+2Rarcsin(\frac{\lambda }{2R}))(-dR)$$

Mathematically dR is defined as the difference between radii of E_2_ and E_1_, which is negative for this bowing configuration. Hence, the negative sign for dR is to ensure a positive value of dR.

It takes more effort to acquire S_2_ since it involves three shaded areas in Fig. 7. S_2_ can be calculated as the sum of blue and orange shaded area after subtracting the area marked in green.

17 $${S}_{2}=f({\lambda }_{2},R)+\frac{\lambda dlsin\theta }{2}-f(\lambda ,R)$$

A similar approximation method is used to solve $f({\lambda }_{2},R)-f(\lambda ,R)$. The result is given as

18 $${S}_{2}=(\frac{R}{\sqrt{4-\frac{{\lambda }^{2}}{{R}^{2}}}}-\frac{2{R}^{2}-{\lambda }^{2}}{2\lambda \sqrt{4{R}^{2}-{\lambda }^{2}}})dlcos\theta +\frac{\lambda dlsin\theta }{2}$$

Up to this point, most of the terms in the energy criteria equation (20) are solved except for the interfacial dislocation energy, E_i_. Results from earlier studies suggest that the energy of interfacial dislocations is lower than that in the matrix^20^. The line energy at the particle interface is given as:

19 $${E}_{i}=\frac{G{b}^{2}}{2}{\rm{{\rm K }}}dl$$

where ${\rm K }$ is the relaxation parameter which is related to the interface property such as coherency and atomic bonding strength. The presence of interfacial dislocations is believed to alter the interface atomic structure locally due to dislocation core relaxation and spreading that lowers the energy of the interfacial dislocation. The value of ${\rm{{\rm K }}}$ ranges from 0 (interfacial dislocations have no energy) to 1 (no interfacial dislocation relaxation). We will use this equation to study how interfaces affect interfacial glide.

Inserting all the energy terms into the energy criteria (7) and simplifying, results in the following equation:

20 $${\rm{{\rm K }}}-sin\theta x+\sqrt{1-{x}^{2}}cos\theta  < 0$$

It should be noted that equation (20) is valid for θ from 0 to 180° even though the schematic in Figs 6 and 7 only shows θ smaller than 90°.

### Model application

For any given precipitate-dislocation configuration, equation (20) can be used to predict whether or not interfacial glide is energetically favorable. For example, if the pinning precipitates are perpendicular to initial dislocation line direction, then θ is set equal to 90°. For this geometry, equation (20) yields the energy criteria that are required to satisfy ${\rm K } < x$. If interfacial glide can occur, equation (20) must solve a value of x within the range from 0 to 1. In the situation above, if no interfacial dislocation relaxation exists in this interface (${\rm{{\rm K }}}$ = 1), there is no resolved x from equation (20) meaning no interfacial glide is allowed. In fact, for ${\rm{{\rm K }}}$ = 1, for any given value of θ we input into equation (20), there is no solution for x. In conclusion, the energy criteria suggest that the root cause of interfacial glide is the existence of interfacial dislocation relaxation.

For any ${\rm{{\rm K }}}$ < 1, interfacial glide may be activated but not necessarily for all precipitate-dislocation configurations. Solving the equation (20) under different constraints provides the conditions under which interfacial glide can occur. The mathematical derivation is omitted.

1. When ${\rm{{\rm K }}}$ < 1, interfacial glide is not allowed if orientation θ is smaller than θ^*^ where
  21 $${\theta }^{\ast }=arccos\sqrt{1-{{\rm{{\rm K }}}}^{2}}$$
2. When ${\rm K }$ < 1 and θ^*^ < θ, interfacial glide happens when
  22 $$x={\rm{{\rm K }}}sin\theta +\sqrt{1-{{\rm{{\rm K }}}}^{2}}cos\theta $$
3. When ${\rm K }$ < 1 and θ > 90°, the left side of equation (20) is always smaller than zero if θ is larger than θ^**^

23 $${\theta }^{\ast \ast }=\pi -arccos{\rm{{\rm K }}}$$

When θ is larger than θ^**^, interfacial glide will occur spontaneously.

Case 1 indicates a threshold value for initiation of interfacial glide. It also implies an orientation-dependent effect. If the precipitate has orientation factor θ smaller than θ^*^, no interfacial glide is allowed meaning the bowing magnitude of the pinned dislocation, $\frac{\lambda }{2R}$, can reach a value of 1. Recall that equation (3) can be written in terms of the bowing magnitude x to reflect the maximum back stress when dislocation reaches the critical condition.

24 $$\begin{array}{rcl}\tau  & = & \frac{Gb}{2R}=\frac{Gb}{\lambda }x\\ \tau  & = & \frac{Gb}{\lambda }({\rm{{\rm K }}}sin\theta +\sqrt{1-{{\rm{{\rm K }}}}^{2}}cos\theta )\end{array}$$

**Figure 8:**
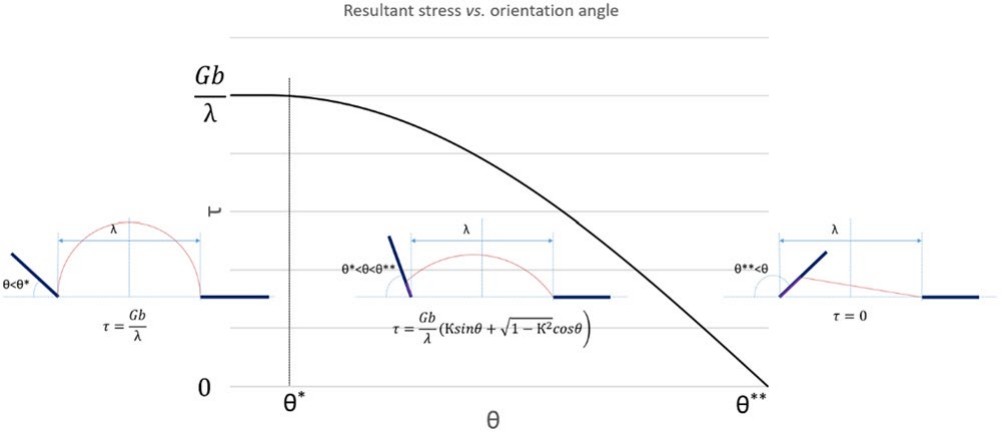
The resultant stress τ is monotonically decreasing because of interfacial glide. As the schematics shows no interfacial glide occurs when orientation angle θ is smaller than θ^*^. The stress is a function of θ when orientation angle is between θ^*^ and θ^**^. The interfacial glide will occur spontaneously when θ is larger than θ^**^.

From Case 2, the resultant stress calculated for the critical condition is a function of the orientation factor θ. It follows that the stress τ is monotonically decreasing with θ when it ranges from θ^*^ to θ^**^. The function of equation (24) is plotted in Fig. 8. This explains the experimental result in section 2*.b* where dislocations are bowed strongly for segment 1 and no interfacial glide is allowed to initiate whereas for segment 2, it is easy for interfacial glide to occur. Because of the different precipitate-dislocation configurations for the two segments, the one with smaller precipitate orientation angle is more difficult or even impossible to initiate interfacial glide and this is the case for segment 1. Although it is not possible to measure the external stress from the *in situ* video, the shape of bowed dislocation can be used to infer the level of stress acting on dislocation. The stronger bowing shape of segment 1 indicates a higher back stress against the external stress, which supports the hypothesis that interfacial glide is sensitive to precipitate orientation and may have an influence on the overall precipitation hardening. In addition, Case 3 provides an interesting situation in which the dislocation segment will spontaneously glide along the interface because the energy criteria is automatically satisfied when θ is larger than θ^**^. This may explain the result in section 2*.b* where the initial incoming dislocation segment 1 directly deposits interfacial dislocations on the first precipitate interfaces. However, this mechanism is only accountable for pining at the leading edge of the precipitate. After the interfacial glide, the dislocations become anchored at the departure end of the precipitate and thus the strengthening also depends on the detachment process. The pinning effect from the departure side is unsolved. For example, dislocation segment 1 in Fig. 1 receive strong pining from the left end resulting in a strong bowing shape whereas segment 2 in Fig. 3 easily glides out of view from departure side without severe bowing. The overall precipitation strengthening is determined by the higher pinning effect from leading and departure end. The detachment process is currently under investigation.

**Figure 9:**
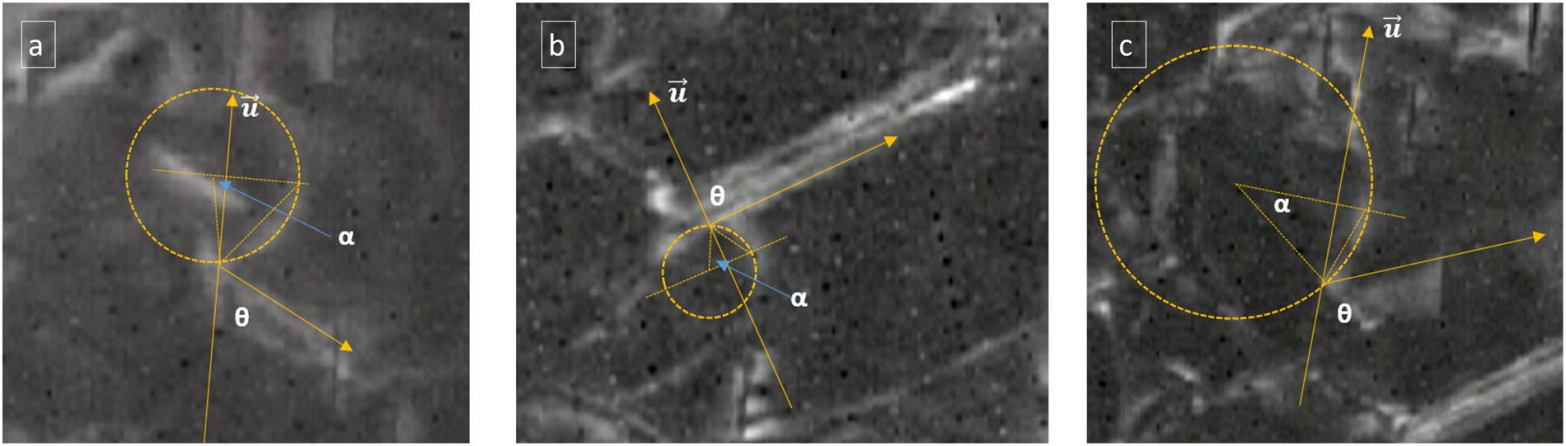
Method for measuring the half central angle for three critical conditions used to determine the relaxation parameter ${\rm{{\rm K }}}$. All images are exerted from *in situ* video recorded in dark field condition with near basal orientations. The yellow arrows show broad facet orientations and the initial dislocation line direction.

As indicated, the interface relaxation parameter ${\rm{{\rm K }}}$ is of great significance in determining if interfacial glide is to be expected. Equation (20) provides a method for determining the interface relaxation parameter ${\rm{{\rm K }}}$. The value of ${\rm{{\rm K }}}$ can be determined if the orientation angle θ is measured from *in situ* experiments and the bowing magnitude is extracted from critical conditions. However, practically, the bowing magnitude is difficult to precisely measure due to the image quality and strain field around dislocations and precipitates. A shape fitting method was used to increase the measurement accuracy. A circle is matched to the curved segment, hence the half central angle α is measured graphically from the critical condition and the bowing magnitude is determined. In Fig. 9, three critical conditions are extracted from *in situ* videos. The first one comes from section 2*.a* and the other two come from another video but all in the same basal view direction. The results are summarized in Table 1. Although the three critical conditions have different precipitate-dislocation configurations, the calculated relaxation parameters are relatively consistent. The average value of ${\rm{{\rm K }}}$ is 0.86. The relative constant value of ${\rm{{\rm K }}}$ suggests that the interface dislocation relaxation is closely related to interface itself, which is thought to be linked to the interface geometry and atomic structure^20^. Research characterizing this relaxation parameter in other alloy systems^20,21^, especially in oxide dispersion strengthened (ODS) alloys suggests that interface dislocation relaxation affects the creep behavior of those alloys. In the ODS systems, the reinforcing particles are incoherent with the matrix. A lower bound for relaxation parameter has been determined to be 0.66 for the superalloy INCONEL MA754^20^. An upper bound value for ${\rm{{\rm K }}}$ of 0.93 has been determined for MA6000^21^. Although this phenomenon is widely observed, the physical meaning remains unclear. In semi-coherent metallic interfaces^22^, dislocation core spreading may lead to interface relaxation. Incoherent or semi-coherent interfaces which have relatively lower shear strength of the interface can ease atomic rearrangement and facilitate local interface diffusion resulting in a lower interface dislocation energy^23^. The value of ${\rm{{\rm K }}}$ of 0.85 in the present work seems to fall into a reasonable range. Although β_1_ precipitates in Mg are generally reported to be coherent^24^, the samples characterized in this study were over-aged and may have resulted in semi-coherent precipitates. The atomistic mechanism to lower the interfacial dislocation energy cannot be interpreted with the current model which is based on linear elasticity, and should be investigated by atomistic simulations.

**Table 1:** Relaxation parameter determination on three different configurations.

	θ/°	α^*^/°	x	${\rm{{\rm K }}}$
a)	62	81.9	0.99	0.81
b)	90	64.2	0.90	0.90
c)	113	36.1	0.59	0.86

The dislocation energy model in this study explains the theoretical foundation of interfacial glide and shows the orientation-dependent effect. Nevertheless, it should be recognized that many simplifications have been made in deriving this model. In reality, dislocation energy is sensitive to Burgers vector and surrounding strain field from other dislocations and precipitates. Calculations related to individual precipitate-dislocation interaction are only suitable for ideal situations. A more accurate study may involve use of dislocation dynamics (DD) simulations. A better understanding of interfacial glide is believed to be important for alloy development. It could be a general mechanism for dislocations interactions with linear extended precipitates. From the analysis of the relaxation parameter ${\rm{{\rm K }}}$, this study suggests that interface engineering may play an important role in determining the strengthening effect due to extended precipitates.

## Conclusions

*In situ* straining in TEM is performed on over-aged Mg-2.35% *wt*.% Nd binary alloy. The unit process for precipitate-dislocation interaction was observed for extended lath β_1_ precipitates. A dislocation based model is developed to analyze the unit process. The key findings from the experimental characterization and theoretical analysis are summarized below.

1. The pinned dislocation may glide along the precipitate broad facet to overcome precipitate blocking, thereby creating interfacial dislocations.
2. An orientation-dependent effect is observed suggesting a stronger pinning effect for precipitates with low orientation angle θ, as defined in Fig. 5.
3. Dislocation theory-based analysis shows that the proposed interfacial glide mechanism is energetically favorable if interface dislocation relaxation exists.
4. The model shows that the resultant stress for glide is affected by interfacial glide which is determined by the orientation angle θ and an interface relaxation parameter ${\rm{{\rm K }}}$ that ranges from 1 (no glide along the interface implying high resistance to creating interfacial dislocation segments) to 0 (easy glide along the interface, low interface resistance to creating interfacial dislocation segments).
5. A higher value of θ and a lower value of ${\rm{{\rm K }}}$ will result in a lower value of the glide stress for single dislocation, and ultimately a reduced level of strengthening from leading edge of the precipitate.

## Experimental Methods

**Figure 10:**
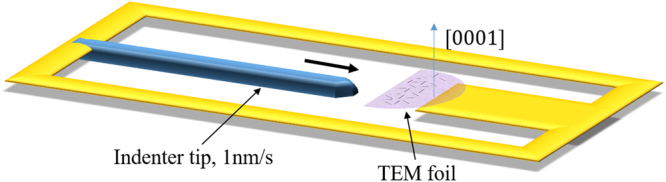
Schematic of *in situ* straining setup. The TEM foil in this figure is selected with its normal (blue arrow) closely parallel to [0001]. Black dashes in foil represent precipitates of three different variants. The indentation direction is indicated by thick black arrow.

An as-cast Mg-2.35 wt.% Nd binary alloy provided by CanmetMATERIALS was used in this investigation. The alloy was prepared by resistance heating in a steel crucible under a protective atmosphere of CO_2_ and 0.5% SF_6_ gas and then poured into a mold with diameter of 3.5 inches. Castings were solution treated at 530 °C for 3 hours and water quenched. The aging condition was 9 hours at 250 °C to ensure that β_1_ was the dominant precipitate. Specimens for transmission electron microscopy (TEM) were mechanically thinned to 100 µm and electropolished following the procedure from Nie and Muddle^25^. The edge of specimen is preferentially electropolished to electron transparency. The electropolished sample was glued to the stage of a Hysitron PI95 TEM Picoindenter leaving the transparent region facing the 1 µm flat punch indenter tip. Figure 10 schematically shows the *in situ* straining setup. Local deformation is introduced by indentation at a displacement rate of 1 nm/s and dislocation activity was recorded by a CCD camera at a frame rate of 25 frames/second. Since the area of the TEM foil deformed by the indenter tip is small with respect to the grain size, the deformed region can be regarded as a part of a single crystal. Most of selected foils have an orientation near basal plane and some near to $(\bar{1}2\bar{1}3)$ plane. Experiments are conducted in a JEOL 2010F transmission electron microscope.

### Data Availability

All data generated or analyzed during this study are included in this published article (and its Supplementary Information files).

## Electronic supplementary material

Supplementary video for 2.a

Supplementary video for 2.b
